# Diagnosing Barriers to California Bay-Delta Wetland Restoration Implementation: Extending the Adaptation Perspective

**DOI:** 10.1007/s00267-026-02618-7

**Published:** 2026-09-11

**Authors:** Kyra Gmoser-Daskalakis, Mark Lubell, Gwen Arnold

**Affiliations:** 1 Work Primarily Conducted At: Department of Environmental Science & Policy, University of California Davis, Davis, CA USA; 2 Department of Environmental Science & Policy, University of California Davis, Davis, CA USA; 3 Present Address: Copernicus Institute of Sustainable Development, Utrecht University, Utrecht, Netherlands

## Abstract

Climate adaptation increasingly considers wetland restoration as a nature-based solution to reduce flood risk and provide co-benefits such as species protection, water resource management, and public recreation access. However, barriers must be identified and overcome to increase the pace and scale of wetland restoration for adaptive benefits. We argue existing climate adaptation barriers frameworks insufficiently address physical and ecological barriers that become apparent during on-the-ground implementation of wetland restoration projects. This paper diagnoses barriers to wetland restoration projects in the California Bay-Delta Estuary via semi-structured interviews with 59 project developers and regional restoration policymakers, funders, and managers across nine case study projects. Findings suggest common challenges emerge around conflicts among regulatory authorities and insufficient funding for post-project stewardship. We recommend potential solutions to address key barriers and increase the pace of wetland restoration. Adaptation in developed coastal estuaries worldwide increasingly requires nature-based solutions like wetland restoration; such a framework is imperative to diagnose barriers and identify interventions that can increase investment in these complex social-ecological systems.

## Introduction

As climate change impacts accelerate, particularly increased flooding and sea level rise, policymakers and scientists push for nature-based solutions as adaptation (Gupta et al. 2024; Chausson et al. 2020; van Rees et al. 2024). Nature-based solutions (NbS) use natural processes and features to address societal challenges like climate change and provide social and environmental benefits (IUCN 2016). Wetland restoration recovers destroyed or damaged wetland habitat (Sapkota et al. 2018); it is a type of NbS because restoration actions create habitat to provide climate adaptation (e.g., flood protection, sea level rise adaptation), biodiversity, water quality, carbon sequestration, and recreation (de Groot et al. 2018). Regional, national, and global environmental plans and policies now require or encourage wetland restoration as a climate adaptation NbS (Hettiarachi et al. 2015; Goals Project 2015; Exec. Order No. 14008 2021).

Despite this policy emphasis, the pace of restoration is insufficient to meet policy goals or even offset continued wetland loss in U.S estuaries (Gittman et al. 2019). To accelerate the pace of wetland restoration, we need to diagnose the barriers to implementation and develop policy recommendations. This problem motivates this paper’s research question: what are the main barriers to implementing wetland restoration projects as NbS solutions? We argue that the two strands of existing literature that target this question have not provided sufficient answers, for different reasons.

The first strand of research is case studies of specific wetland restoration (Bell-James 2022; Clare and Creed 2022; Sayles 2018) and nature-based solutions programs (Raska et al. 2022; Sanchez-Arcilla et al. 2022; Stewart-Sinclair et al. 2020; Tuihedur Rahman et al. 2021). While the case study literature documents barriers for specific projects or locations, it has not developed a generalizable approach that can then be applied to future cases. The second strand is general frameworks for classifying barriers to climate adaptation actions (Biesbroek et al. 2013; Moser & Ekstrom 2010). These general barriers frameworks are insufficient because they mostly focus on regional policy or planning activities and do not adequately account for on-the-ground realities of implementing projects that functionally achieve adaptation goals. Further, few studies propose solutions to identified barriers (Sayles 2018; Deely et al. 2020; Bataille et al. 2023; Schultz et al. 2019; Moser and Ekstrom 2010). In other words, general barriers frameworks do not delve deeply enough into the implementation process.

To address this conceptual problem, we integrate and adapt two climate adaptation barriers frameworks to incorporate physical and ecological site conditions. Our approach diagnoses barriers and illuminates solutions to accelerate implementation, providing lessons for estuaries worldwide with wetland restoration policy goals. We apply our framework to the case of the California Bay-Delta (Bay-Delta), a highly-developed estuary where policymakers have long sought to restore the 85-90% of wetlands lost to agriculture and development (USGS 2017; Orr 2018), and now pursue wetlands restoration as a nature-based solution to climate change impacts like coastal flooding and sea level rise (Goals Project 2015; Bay Adapt 2021; SFBRA 2023). Regional plans set ambitious restoration targets (i.e., 100,000 tidal marsh acres in the Bay, 60–80,000 habitat acres in the Delta) (Goals Project 2015; DSC 2022). We identify barriers and potential solutions to Bay-Delta wetland restoration using interviews with developers of nine case study projects and regional restoration actors who shape the local context. We find key barriers from regulatory conflicts, on-site constraints, and funding.

The remainder of this paper is organized as follows. We set the stage for the analysis by describing the evolution of wetland restoration from ecological goals to climate adaptation in our case, the Bay-Delta estuary. We then provide an analysis of the existing literature and propose an analytical framework for diagnosing barriers that addresses the gap in existing research. The method section describes how we qualitatively code key informant interviews from nine Bay-Delta wetland restoration projects to diagnose key implementation barriers, which provides the basis for potential solutions outlined in the discussion section.

## Background: From Wetland Restoration to Climate Adaptation

**Fig. 1:**
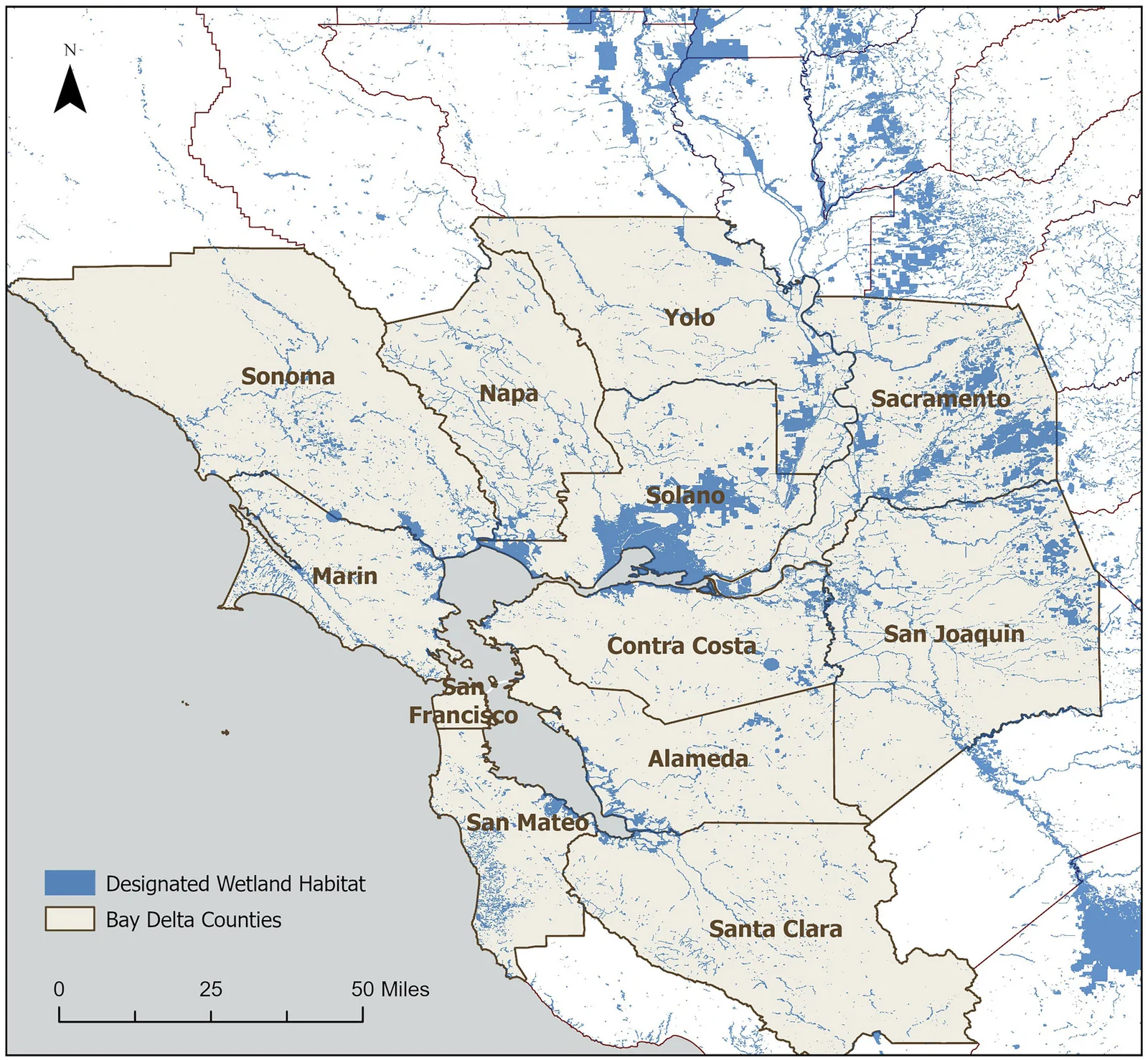
Bay-Delta Counties with Existing Wetland Habitat per CA Nature Habitat and Land Cover Designation (CA Department of Technology 2024, CEC 2024)

The Bay-Delta estuary extends from the Sacramento-San Joaquin Delta, where freshwater flows through the brackish Suisun Marsh, out to saline San Francisco Bay (Fig. 1). The Bay-Delta is formed by the convergence of Sacramento and San Joaquin rivers, which receive water from the Sierra Nevada mountains. The estuary provides critical species habitat, water resources for much of the state, and extensive urban development and agricultural production (U.S. EPA 2021; State Water Board 2018). The Bay and Delta have complex and overlapping local, state, and federal authorities with competing goals for water, habitat, and development (Lubell et al. 2013).

For at least 50 years, project developers have pursued wetland restoration in the Bay-Delta (Orr 2018). The 1965 federal McAteer-Petris Act limited filling of San Francisco Bay wetlands. The Clean Water Act established compensatory mitigation requirements, leading to increased marsh restoration in the 1980s–1990s (Orr 2018). The complexity and size of wetland restoration projects have grown over time; ongoing restoration represents a mix of voluntary projects and legally-required mitigation to offset habitat loss and environmental impacts of other activities (Callaway et al. 2011; Orr 2018).

Bay-Delta restoration is increasingly motivated by climate adaptation goals for flooding and sea level rise. Updates to Baylands habitat restoration goals added sea level rise considerations (Goals Project 2015) and more recent Bay-Delta climate adaptation plans (Bay Adapt 2021; DSC 2022) and restoration funding guidelines (SFBRA 2023) frame wetland restoration as a climate adaptation NbS. Wetlands buffer storm surges and reduce peak flows, providing flood protection alongside other co-benefits (Saleh and Weinstein 2016; Chausson et al. 2020; Tuihedur Rahman et al. 2021).

Yet wetland restoration remains insufficient to achieve regional targets. Separate plans in the estuary call for 100,000 acres of tidal marsh restoration in the Bay (Goals Project 2015), 5–7000 acres of tidal restoration in the Suisun Marsh (SMP 2013), and 60–80,000 acres of Delta habitat restoration (DSC 2022). Meeting these goals of up to 187,000 restored acres requires significant increase in current restoration; only 16,400 acres of Bay tidal restoration occurred from 1998 to 2020 (WRMP et al. 2025) and 13,758 acres of Delta wetland restoration is completed or in-progress from 2007–2023 (Austin 2023). Identifying barriers to implementation and potential solutions is essential to increase the pace of restoration. We next consider how existing climate adaptation barriers frameworks apply to wetland restoration.

## Theoretical Background: Barriers to Wetland Restoration

Wetland restoration research faces a conceptual dilemma; on the one hand, individual case studies that consider site-level barriers in implementation have not been synthesized into a generalizable framework that applies broadly. On the other hand, replicable frameworks based in climate adaptation literature do not adequately incorporate project-scale considerations (OnlineResource1).

Previous studies examine governance barriers in coastal NbS (Dawson et al. 2017; Sayles 2018; Tait & Brunson 2022; Velez et al. 2018; Raska et al. 2022; Stewart-Sinclair et al. 2020; Cortina-Segarr et al. 2021; Sanchez-Arcilla et al. 2022; Bell-James 2022; Bell-James et al. 2023). Each study identifies barriers with its own classification system, often focused on higher-scale governance and planning with less attention to on-the-ground implementation. Fewer studies on barriers to NbS or wetland restoration integrate project and regional scales, though some consider legal and land ownership barriers alongside financial, technical, and governance barriers (Bell-James 2022; Cortina-Segarr et al. 2021; Raska et al. 2022). Sayles (2018) uses interviews to understand barriers in wetland restoration at the regional- and project-level over time (for Puget Sound, Washington). Clare & Creed (2022) identify barriers to wetland restoration on private lands in Alberta, Canada and find barriers from interactions of environmental, social, economic, and political factors on-the-ground. We respond to their call to focus more on restoration practitioners.

Generalizable frameworks exist, but lack this focus on project-scale interactions of social, environmental, and political factors. Biesbroek et al. (2013) and Moser and Ekstrom (2010) are two replicable, generalizable frameworks for classifying climate adaptation barriers. Biesbroek et al. (2013) define barriers as factors or conditions perceived to negatively affect adaptation processes or outputs. They outline five categories for climate adaptation barriers: Cognitive, Financial, Informational, Institutional, and Social. They find the adaptation literature most commonly identifies institutional and social barriers; Biesbroek et al. (2013) does not include a category for physical or ecological conditions. Another blue-green infrastructure barriers framework does include on-site limitations, but does not consider temporal or spatial scales of actors and project processes (Deely et al. 2020). The temporal and spatial nature of restoration creates unique barriers; projects take place over long periods of time in varying locations with both site-specific and landscape-level social and ecological factors. Moser and Ekstrom (2010) diagnose barriers to climate adaptation by identifying the spatial and temporal scales at which barriers originate. Existing barriers frameworks each have shortcomings in translating to restoration; they either fail to account for physical and ecological conditions or the spatial and temporal scales involved in project processes.

**Fig. 2:**
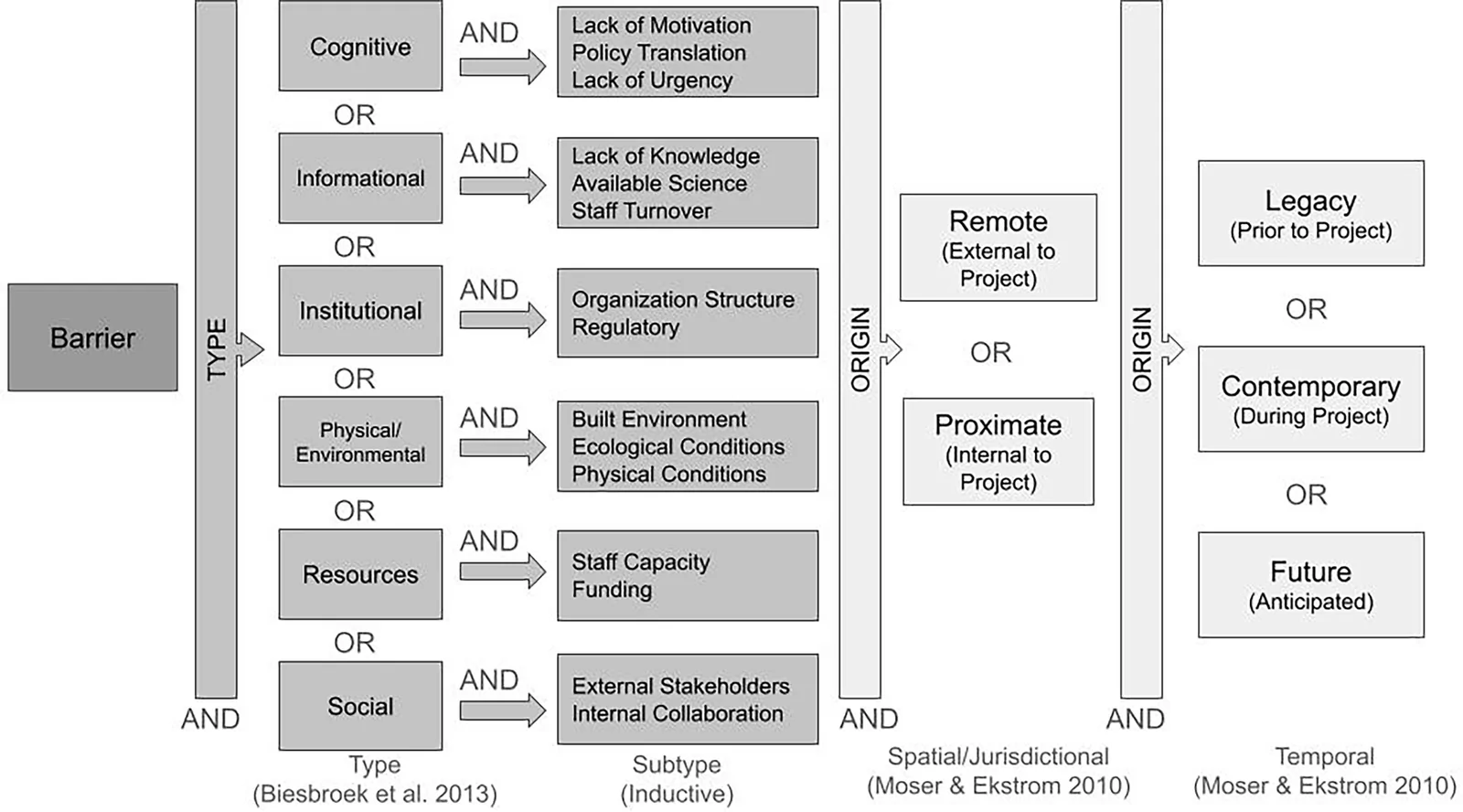
Framework to Diagnose Barriers to Wetland Restoration

To address both the lack of generalizable frameworks in wetland-specific barrier studies and the insufficient treatment of restoration realities in generalizable adaptation barriers frameworks, we integrate and update the Biesbroek et al. (2013) and Moser and Ekstrom (2010) frameworks for wetland restoration. In our framework, a barrier to a wetland restoration project is categorized by what the barrier is (type, subtype) and where (spatial origin) and when (temporal origin) it emerges relative to the project (Fig. 2). Our additions of a physical/environmental barrier type and a future temporal origin better capture site-specific realities that constrain restoration implementation. This framework can help identify solutions to increase wetland restoration implementation to meet policy goals.

## Data and Methods

To analyze wetland restoration barriers in the Bay-Delta, we conducted semi-structured interviews with partners of nine projects and regional actors (policymakers, funders, program managers) who fund, regulate, or implement wetland restoration.

### Case Study Project Selection

We select projects from EcoAtlas, an online database of environmental projects across California recognized as the region’s most comprehensive inventory of restoration (CWMW 2024). Each project entry contains—where provided by partners or database managers—project location, type, status, size, habitat, and budget, and the organizations funding or implementing the project^1^. After filtering for entries within Bay-Delta counties (Fig. 1), we selected nine projects for sufficient diversity and coverage across geographic location, years, project partners, size, habitat, and funding sources. These projects cover over 25,000 acres and count toward current regional targets (WRMP et al. 2025, Austin 2023). Conversations with four regional experts (two Bay, two Delta) confirmed the cases are a representative sample of Bay-Delta wetland restoration.

**Fig. 3:**
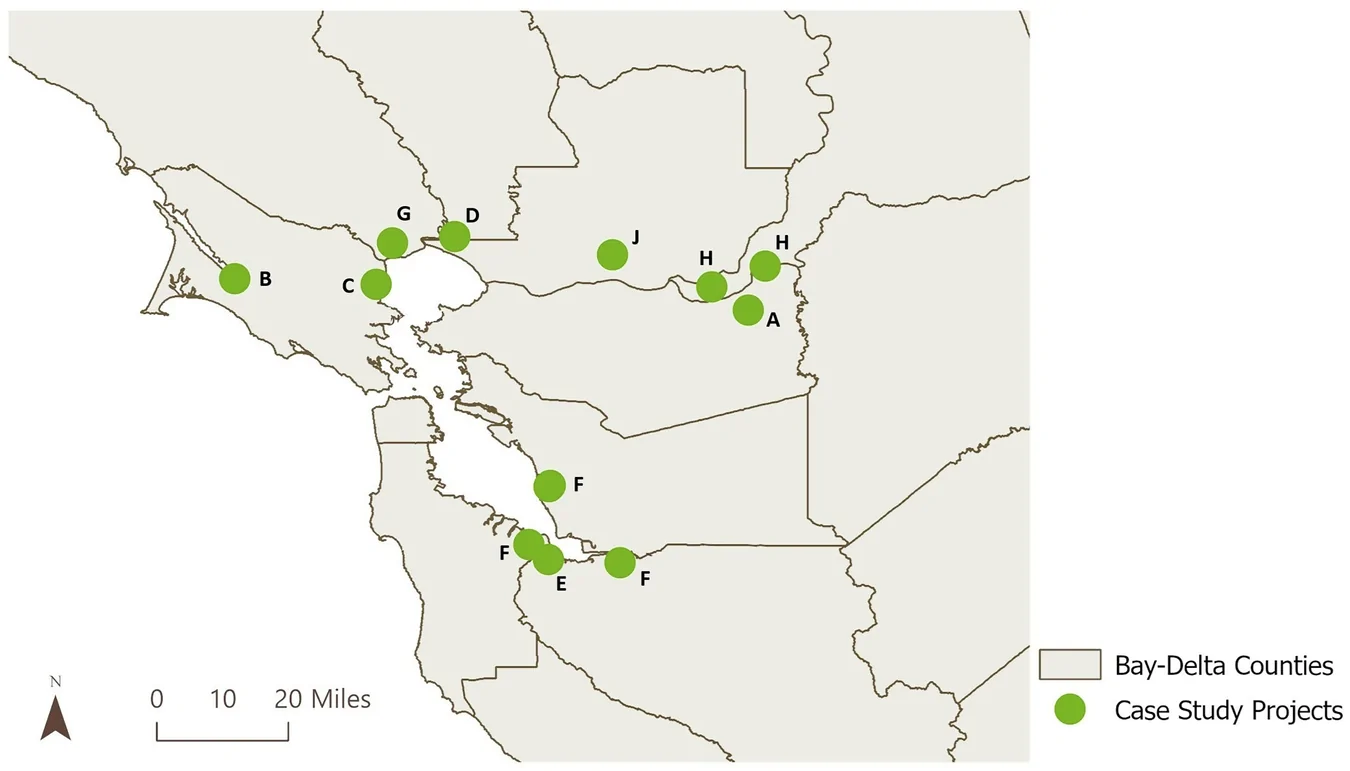
Map of Case Study Projects

The nine case studies span the Bay (6) and the Delta (3) (Fig. 3, OnlineResource2 for case details). One project is in initial planning/design stages (E) with two ongoing (i.e., partial implementation) (A, F), and six completed (B, C, D, G, H, J). Seven projects are voluntary (B, C, D, E, F, G, H) and two were built for mitigation requirements (A, J). Three are brownfield sites of former industrial or military use (C, D, F). Four sites restore former farmland (A, B, G, H). Site J converted managed wetlands for duck hunting to tidal wetland. Site H involves multiple related projects across two sites for subsidence reversal, carbon sequestration, levee improvement and habitat. The first author toured four case study sites in-person (C, E, F, G) and conducted background research of public project documents for project information. Internet searches of project names identified project documents (environmental review, monitoring or feasibility studies, adaptive management plans), news stories, blogs, project websites, presentations (conferences, public meetings), and regional plans/reports.

### Semi-Structured Interviews

Interview questions asked about project benefits, goals, timelines, collaboration, and barriers (OnlineResource3). Project partner participants held roles directly related to project implementation: project managers, engineers/designers, biologists/scientists, construction managers, and community outreach specialists. Regional actors included funders, politicians, policy coordinators, and environmental program managers. Initial participant identification used EcoAtlas (each project entry contains 2–3 contacts) followed by snowball sampling. We sent 76 requests^2^ and conducted 56 interviews with 59 participants^3^ in two waves (September 2023–February 2024, July-December 2024). Participants provided informed oral consent consistent with human subjects ethics approval granted by UC Davis Institutional Review Board (#2008937-1). Four to seven participants represented each project; an additional 14 interviews with regional restoration actors do not tie to a specific project. Of the 59 participants, 23 work in the Delta and 36 in the Bay (Table 1). Participants represent the diversity of governmental, non-governmental, and private organizations involved in wetland restoration.

**Table 1:** Overview of Interview Participants

	Interview Participants (*N* = 59)
Location	23 Delta, 36 Bay
Job Title	Technical Staff (24), Project/Program Managers (22), Policy Actor (13)
Organization Type	Federal Agency (5), NGO (8), Partnership or Regional Government (4), Private (10), Local Government or Special District (10), State Agency (21), University (1)
Project Case Studies	A (5), B (5), C (7), D (4), E (4), F (5), G (4), H (7), J (4), Regional (14)

Interviews lasted 25–71 min (Average: 43 min) on Zoom videoconferencing (44 interviews), in-person (5) and by phone (7). Zoom transcribed online interviews while Otter.AI transcribed phone and in-person recordings. The first author and a student assistant cleaned all transcripts for accuracy.

### Barrier Statement Coding and Analysis

Review of each interview transcript identified statements meeting the Biesbroek et al. (2013) definition of a barrier (factors or conditions the participants perceived to negatively affect the process or its success). The first author manually selected and coded 829 barrier statements^4^ in *Atlas.ti* software (the software only facilitated exporting codes for analysis). Most barrier statements resulted from one interview question: *What were the main barriers you experienced on the project*^5^*?* But transcripts were reviewed for statements in full because participants mentioned barriers throughout interviews (OnlineResource3). Statements and codes were merged with participant and case study details^6^. Each statement refers to a single barrier; participants made 2–34 barrier statements each (Average & Median: 14) with an average of 69 barriers per case study (OnlineResource6). The first author assigned each barrier statement (*N* = 829) one type category and one of 15 iteratively coded subtypes (2–3 per type) developed by the first author and reviewed by the second author. Two student assistants coded selected statements to determine Intercoder Reliability^7^.

This study adapts the categories of Biesbroek et al. (2013) to code barrier types (Table 2). Four of the original five categories were used: Cognitive, Informational, Institutional, and Social. The fifth original category ‘Financial’ was renamed to ‘Resources’ to include organizational resources such as staff capacity. A new sixth category (Physical/ Environmental) adapts the framework to restoration. The barrier statements were then coded for the spatial/jurisdictional and temporal origins of each barrier, using an adjusted version of the Moser and Ekstrom (2010) framework (Table 3).

**Table 2:** Barrier Types (adapted from Biesbroek et al. 2013)

Barrier Type (from Biesbroek et al. 2013 unless specified)	Description	Subtypes
Cognitive	Personal perceptions of risk of or need for wetland restoration that limit action among project partners or external actors (e.g., permitters, public)	- Lack of Motivation (lack of leadership/commitment to fund, maintain, support restoration) - Translation of Policy to Implementation - Lack of Urgency (too slow)
Informational	Lack of knowledge, scientific uncertainty or changing science, staff turnover	- Knowledge (lack of) - Science (uncertainty, communication) - Staff Transition/Turnover (knowledge transfer or loss among project partners or regulatory agencies)
Institutional	Challenges with policy, legal, or institutional structures (e.g., regulation or permit design) in project partner organizations or regulations	- Organization Structure (e.g., internal siloes, org protocols, guidance) - Regulatory (e.g., permits, legal, jurisdictional authority)
Physical/ Ecological (added by authors)	Barriers from physical or environmental conditions or site constraints	- Built Environment (e.g., development pressure, utilities) - Ecological (e.g., species, invasive weeds) - Physical Environment (e.g., climate change, weather, soil conditions, erosion)
Resources (renamed from Financial in Biesbroek et al. 2013)	Financial issues around funding and lack of staff resources (capacity or staff time of project partners or regulatory agencies)	- Staff (e.g., capacity, time) - Funding (e.g., timing, combining sources, insufficient for long term maintenance or planning)
Social	Barriers from internal collaborative dynamics among partners or externally with stakeholders (conflicting goals, lack of trust/understanding)	- Stakeholders (external actors including community, adjacent landowners) - Collaboration (internal partner dynamics)

**Table 3:** Barrier Origins (adapted from Moser and Ekstrom 2010)

Origin Scales (from Moser and Ekstrom 2010)	Description/Definitions of Barrier Origin Types
Spatial/Jurisdictional	Proximate- barrier from project site conditions, project partners, or adjacent/affected community (e.g., landowners, utilities, public) Remote- barrier from higher-scale actors external to the project (e.g., regional or state regulators, politicians, or funders) or regional conditions (e.g., landscape-scale climate impacts)
Temporal	Legacy- barrier from pre-existing or prior choices, decisions, or actions (e.g., organization protocols, regulatory policies, prior development or infrastructure placement) Contemporary-barrier arises during the project process (e.g., negotiation with regulatory agencies, outreach, construction) Future- barrier from anticipated actions on the site (e.g., long term maintenance) or future conditions that affect the project or site (e.g., climate change). *Added by the authors*.

For where or with whom a barrier originates, we use Moser and Ekstrom (2010)’s spatial/jurisdictional levels; barriers can be internal or external to the project. ‘Proximate’ barriers result from the project site or project partners. ‘Remote’ barriers originate from higher scales: regional actors, governance institutions, or wider ecological context such as landscape and climatic changes.

For when a barrier originates, we adapt the temporal origins of Moser and Ekstrom (2010). Barriers to wetland restoration may result from factors before, during, or after the project process^8^. Barriers may be ‘Legacy’ (from past decisions or context), ‘Contemporary’ (during the project process) or ‘Future’. We add the ‘Future’ category to the original framework for barriers from anticipated future scenarios (e.g., changing funding priorities, climate change impacts) affecting restoration already underway. These temporal and spatial origins can identify the relevant scales at which to intervene.

Our methods include descriptive statistics of reported barriers and non-parametric tests of association to test for statistically significant differences in barrier types and origins by project and participant characteristics (Bay or Delta, Participant Role, Project). We use Chi-Square tests, except when small sample sizes require Fisher’s Exact Tests (if expected frequency in at least once cell is less than five). All statistical analysis was conducted in *R*.

## Results: Reported Barrier Frequencies and Comparisons

### Frequency of Barrier Types with Examples

**Fig. 4:**
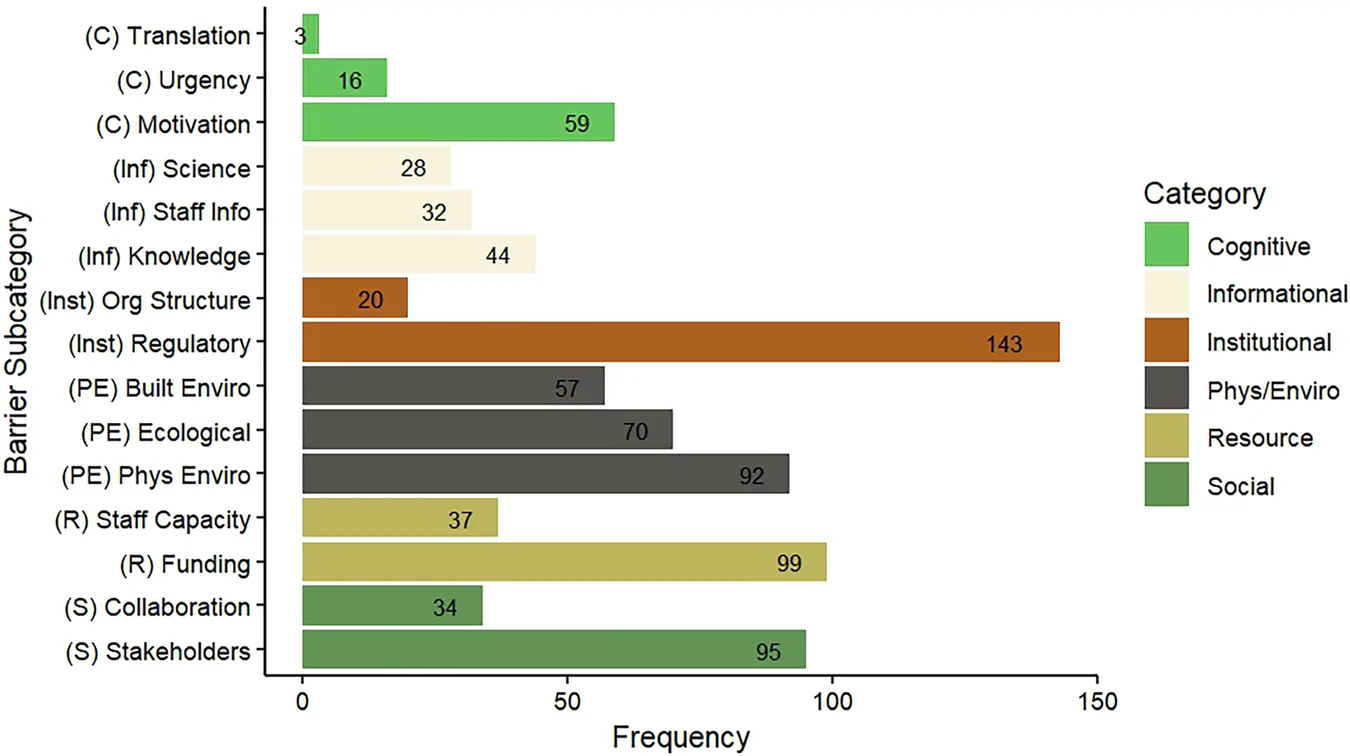
Reported Barriers to Wetland Restoration by Subtype (*N* = 829)

The most common barrier type is Physical/Environmental (219 statements, 26%), reflecting the importance of site-level physical and ecological factors in wetland restoration (Fig. 4). Physical barriers like site elevation, water salinity, or precipitation variability are most common (92, 11%), followed by ecological barriers (70, 8%) such as invasive weeds. But barriers also result from the built environment (57, 7%) given existing infrastructure crosses many land parcels for restoration. Thus, potential conflicts exist between wetland restoration and the management of climate impacts to critical infrastructure.

Built environment barriers are common in developed estuaries like the Bay-Delta, where existing land use constrains potential restoration options. Project implementation to restore habitats depends on site conditions. Participants repeatedly mention infrastructure (e.g., pipelines, railroads) cuts through available land and constrains potential design, while creating regulatory and social barriers from required easements and collaboration with utilities:

“Utilities come up really often in wetland projects. Gas lines, […] high voltage power lines […] wastewater pipelines, water pipelines. So often there’s these large utilities that just go right through areas of historic tidal marshes that we want to restore […] new projects tend to have more challenging constraints to work around” -- R1

“railroad tracks encircle the bay, […] and they’re right in former tidal wetlands. So they’re preventing not only restoration of wetlands, but they’re preventing wetland migration.” -- R6

“from a construction standpoint for implementing new projects […] existing infrastructure is a gigantic hold up […] either you build around them if you can, or […] you can’t build anything here.” -- R26

Participants note that extensive development in the Bay-Delta limits land for restoration. ‘Pristine’ sites without physical constraints from infrastructure or development rarely exist, and not in sufficient quantity to meet restoration goals. This mirrors barriers to other environmental infrastructure; Borgström et al. (2006) argue that challenges to green space implementation are more common in urban landscapes partly due to ‘locked-in’ intensive land uses. Some participants note the literal acreage available to project developers does not match the ‘paper’ acreage called for in regional targets or plans:

“how to actually really achieve acquiring that amount of restoration property when it’s not physically available. It’s a great grand scheme when you put it in writing. But then, as you’re implementing it over the long term, that land availability is not there.” -- R29

“How many years have we been doing restoration, and all the good, easy locations have been taken […] you’re looking at what’s left.” -- R40

But governance challenges matter too; Institutional (20%, 163) and Resources (16%, 136) barriers are second and third most common. Institutional barriers are overwhelmingly from the regulatory subtype (143 of 163, the most common subtype for all barriers). These barriers are from state and federal permits, other legal requirements, local easements, and jurisdictional conflicts over regulatory authority. As one participant noted:

“a lot of the local approvals like getting easements from cities, from flood control districts, having so many different people have to review every single design feature, and then have differences of opinions of what needs to be done. […] It can almost feel like you get frozen in what you can do.” -- R30

Participants often experienced conflicting requirements for different approvals. Some permit requirements for species habitat conflict with public access requirements (CDFW n.d.; BCDC 2005), because sensitive species and human activity are not always compatible (Zedler & Leach 1998; Zingraff-Hamed et al. 2018). Conflicts may occur from distinct habitat preferences of different legally-protected species. Some projects must balance restoring tidal wetlands, which are habitat for endangered species like the Salt Marsh Harvest Mouse and Ridgeway’s Rail, with enhancing managed ponds that provide habitat for protected birds such as Snowy Plover (Palinkas et al. 2022). State regulations require mitigation for habitat impacts if conversion of existing habitats occurs; restoration of wetlands on former agricultural land can require mitigation for the loss of foraging habitat that agricultural fields provide to some species (DWR 2020; Hull et al. 2020). Participants mentioned conflicting species requirements drive up restoration costs and require additional land that may not be available:

“There’s also the permitting aspect, which really can be challenging, to have to do mitigation for a restoration project. You’re trying to build a habitat, and you still have to mitigate for something” -- R31

“So even though you’re providing an ecological uplift, you’re having a negative impact to a listed species. […] having to mitigate for an enhancement project is expensive […] when you’re already trying to do restoration for the good of restoration […] requiring this mitigation, which drives up the costs, and in some cases makes it infeasible to move forward with these projects.” -- R40

Higher restoration costs, including from regulation, creates funding barriers. Resource barriers are largely financial (99 of 136) rather than from staff capacity (37). Funding encompasses varied issues with timing, consistency, flexibility, and requirements. Most common is lack of long-term monitoring or maintenance funding, timing issues in disbursement, expiration/deadlines, and overall insufficiency of funding. Participants repeatedly cited a lack of long-term maintenance funding:

“people’s goals are minimize costs because we can’t afford this. […] you’ve got to manage these areas. You cannot literally just sit and go, Okay, we’re gonna breach it to the tides and we’re done with it. […] you can’t do that.” -- R22

“There is a huge disconnect […] if we’re going to do tidal restoration, there needs to be a commitment of resources for interim management of the site, as well as post restoration, a commitment to manage the site.” -- R23

“most of the funding that’s available is for capital projects; there’s very little funding available for collaborative capacity. […] you have [to have] the partnerships in place first […] that comes out of deep listening, and that is constrained by funding to have relationships.” - R19

Participants mentioned funders may desire ‘no-cost’ restoration or want to ‘walk away’ and ‘let nature take its course’, but restoration entails landscape modifications in an already altered environment and needs stewardship to maintain quality habitat (Moore & Rutherfurd 2017; Galatowitsch & Bohnen 2021; Palinkas et al. 2022). Participants need more long-term funding to manage invasive weeds, adjust water levels, or shift plant compositions over time for future climate and salinity changes.

Other barriers types are less commonly reported. The Informational category (13%, 104) has barriers in knowledge (44), staff transitions (32), and available science (28). Participants noted decades of wetlands science, pilot projects, and regional policy generated practical and scientific knowledge; site-specific, project process barriers were reported more. Social barriers (16%) were more often reported from external stakeholders (e.g., community, regulators, adjacent landowners) (95) than internal project partners (34). Participants discussed difficulties in getting external actors like utilities and regulators to adjust to site-specific realities:

“The [Utility] has been super challenging to work with […] they are not necessarily an active partner who is willing to make compromises and talk about what is needed” -- R30

“working with landowners, working with funders, working with project partners, we’ve all been able to work out those differences and really lead multi-benefit projects. Where it gets a little trickier is with regulatory agencies who have particular mandates and have particular laws that they’re required to have inform all of their work.” -- R30

Different actors in the Bay and Delta make it difficult to create estuary-wide strategies and share lessons learned. Prior studies find Bay and Delta separation creates challenges for water and estuary management (Hanemann & Dyckman 2009; Keeley et al. 2022; Layzer 2013). Participants recognized this disconnect between Bay and Delta restoration communities as a barrier to coordinated, regional restoration strategies:

“There’s very limited interaction between people in the Delta, and people in the Bay. […] it’s also hard because we don’t do it together, an estuary-wide scope.” -- R22

“the separation between the Bay and the Delta is really frustrating for a lot of people […] we’re so separated. It’s like two different worlds.” -- R12

Cognitive barriers are least commonly reported (9%, 78) but often result from a lack of motivation from actors responsible for funding, maintenance, or political decision-making that supports wetland restoration projects. One participant described difficulties getting funders and policy actors to understand the necessity of ongoing land stewardship:

“looking into the future, […] how are we going to maintain it? And how are we going to pay for that? […] So getting people to understand that and commit to this long term management and maintenance of these sites […] that’s certainly the biggest challenge” -- R21

### Barrier Origins

Realities of implementation create proximate (site-level) and contemporary (during the project process) barriers. Slightly more reported barriers are proximate (53%, 439 barriers) than remote (47%, 390 barriers). Site-specific barriers highlight that local project context strongly affects wetland restoration. Resources and Institutional are the most common remote barriers, suggesting current policy, governance, and funding strategies do not adequately meet project-level needs. In timing, the majority of barriers are contemporary to the project process (70%, 581 barriers). Only 16% (133) of reported barriers are legacy, originating from prior conditions or decisions, and 14% (115) from future conditions. This underscores the importance of an implementation focus; project developers experience barriers in the project process not sufficiently considered in regional policy-making or funding. The spatial and temporal origins of reported barriers can help identify where restoration challenges arise and where to intervene. Similar proportions of barriers tend to originate at both proximate and remote scales across time periods (Table 4); in general, most barriers are contemporary during the project process.

**Table 4:** Barrier Frequencies for Combinations of Barrier Origins

	Legacy	Contemporary	Future
**Proximate**	67 (8.1% of all barriers)	316 (38.1% of all barriers)	56 (6.8% of all barriers)
**Remote**	66 (8.0% of all barriers)	265 (32% of all barriers)	59 (7.1% of all barriers)

**Fig. 5:**
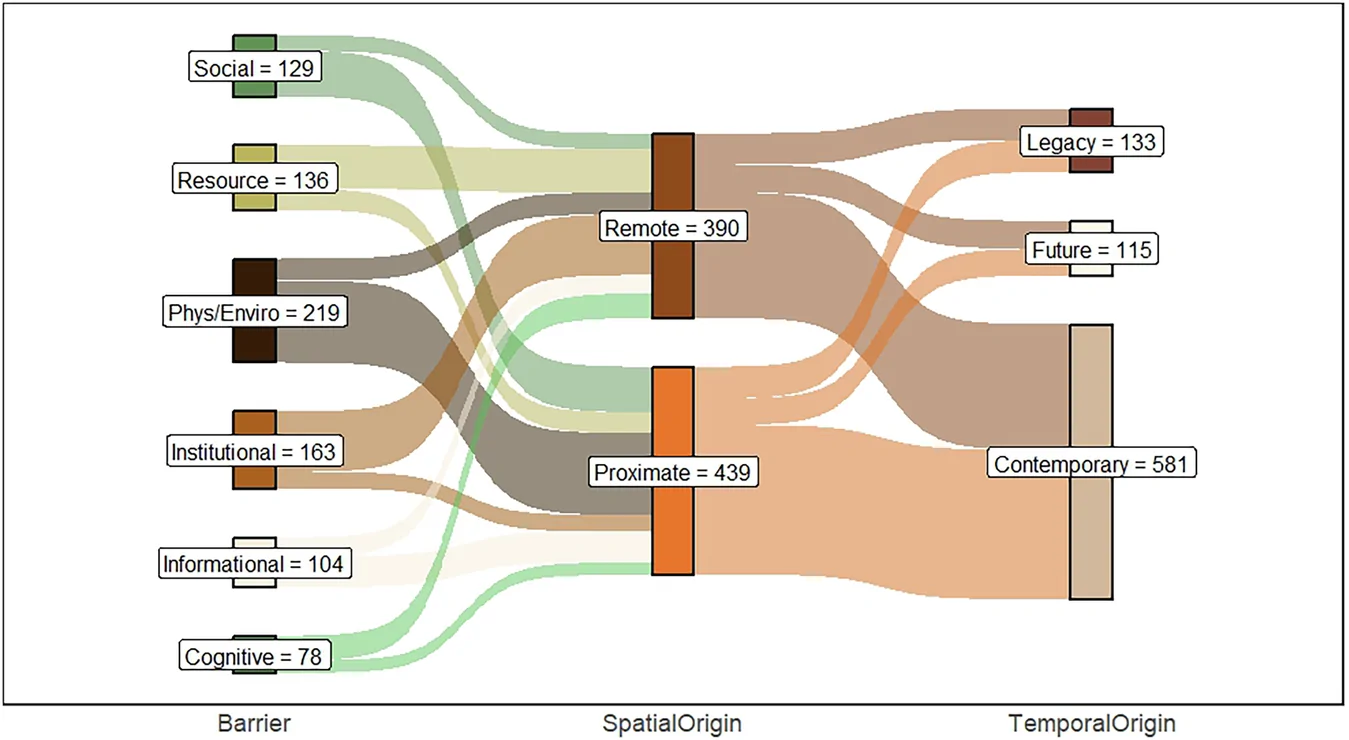
Reported Barriers (*N* = 829) by Type and Origin. The width of flows in the Sankey diagram visually depict the reported barriers by type with different spatial origins (left), and the reported barriers by spatial origins with different temporal origins (right)

However, spatial origins do differ by barrier type. The left hand side of Fig. 5 shows that Physical/Environmental barriers are overwhelmingly proximate (at the project site) while Institutional barriers result more from remote actors and conditions (at the regional or landscape scale). Addressing barriers in these categories may require policy interventions at different governance scales with different system actors. But the right hand side of Fig. 5 shows that most barriers, regardless of spatial scale, are contemporary (arising during the project process). This underscores the importance of considering the project process and site when diagnosing barriers to wetland restoration. But barriers do originate at all scales; accelerating wetland restoration requires multiple interventions with different actors and timing.

### Barrier Comparisons by Location, Project, and Participant Roles

Overall frequencies of barriers may obscure differences in projects within the Bay-Delta. We use non-parametric tests of association (Chi-Square, Fisher’s Exact, see Methods) to test for statistically significant differences in the proportion of barrier types and origins by location, project, or participant type (OnlineResource4).

Despite the ecologically-interconnected estuary, the Bay and Delta differ in regulatory requirements, physical conditions, and landowners. Designs also differ across projects, with goals based on habitat type, stakeholders, and site conditions. Thus, we might expect the most common barriers to differ by project and location. However, differences in barrier type frequencies between Bay and Delta are not statistically significant (*X*^2^ = 9.964, *p*-value = 0.076; OnlineResource5). Participants experience common barriers across the estuary. Bay and Delta barriers are also not significantly different for Spatial/Jurisdictional (*X*^2^ = 0.878, *p*-value = 0.349) or Temporal (*X*^2^ = 4.957, *p*-value = 0.084) origins. Contemporary barriers during the project process dominate across the estuary.

The diversity of reported barriers does reflect unique site conditions; differences in barrier types between the nine projects are statistically significant (Fisher’s Exact Test *p*-value = 0.001) (OnlineResource6). Partners of Project E reported no Physical/ Environmental barriers, likely because the project has yet to reach construction. Project J partners reported fewer Resource barriers; they get dedicated state agency funding as legally-mandated mitigation for environmental impacts of state operations. Differences in Spatial/Jurisdictional barrier origins by project are significant with a Chi-Square test (*X*^2^ = 44.16, *p*-value = 1.319 × 10^-6^). However, differences in temporal barrier origins by project are not significant (Fischer’s Exact Test *p*-value = 0.215). All projects have more proximate than remote barriers, except for Project F which is a large, long-running restoration program (OnlineResource7). Project F partners may engage more remote stakeholders or experience more regional policy and funding changes over time, which could make remote barriers more salient or frequent. Our framework’s ability to identify site-specific barriers across multiple project contexts suggests its potential as a replicable framework to diagnose wetland restoration barriers at project-scales.

We also compare barrier types and origins across three participant role types: project or program managers (25 participants), technical staff (e.g., scientists, construction managers, engineers) (24), and policy actors (e.g., funders, executive directors, community outreach, politicians) (10). While Institutional, Resources, and Physical/Environmental are top 3 barrier types for all roles, barrier types are significantly different by participant role (*X*^2^ = 27.081, *p*-value = 0.003). ‘Policy Actor’ participants reported more Resource barriers and fewer Physical/Environmental barriers than project-level participants (OnlineResource8). Project managers and technical staff are the frontline for design, engineering, and construction decisions and directly experience site conditions more than external policy actors. Spatial/Jurisdictional origins are also significantly different by role (*X*^2^ = 6.999, *p*-value = 0.030). ‘Policy Actors’ reported more remote barriers while project managers and technical staff reported more proximate barriers (OnlineResource9). Site-level challenges are highly salient to project partners. However, differences in Temporal origins by role type are not significant (*X*^2^ = 4.812, *p*-value = 0.307). The implementation phase was a key locus of barriers for all participants. Different barrier experiences among different stakeholders suggests the need for collaboration across the wetland restoration governance system, in order to integrate policy actors and project managers with diverse knowledge and resources. We consider changes in collaboration, alongside permitting and funding, to accelerate Bay-Delta wetland restoration.

## Discussion: Current Efforts and Potential Solutions

For participants, barriers most often arise from conflicts or tensions between the project scale and the scale of regulation and funding. We explore three of the most common barriers around regulation, built environment, and funding which reflect these tensions and recommend strategies to strengthen current efforts to address these barriers in the Bay-Delta.

### Addressing State and Federal Regulatory Barriers

Participants most commonly report barriers in the regulatory subtype, justifying California’s increased focus on streamlining regulatory processes for restoration. Current state efforts include the “Cutting Green Tape Initiative” (CGT) by the California Natural Resources Agency and simplified state and federal permits for restoration (TNC 2024). As part of CGT, the California Department of Fish and Wildlife (CDFW) consolidated authorization into fewer permits, the State Water Resource Control Board developed a statewide order for water quality certification, and CDFW now exempts restoration projects of certain sizes and types from California Environmental Quality Act (CEQA) review (CNRA 2022). U.S. Fish and Wildlife Service (USFWS) has a statewide biological opinion for restoration, meaning California restoration projects need not undergo individual review for threatened or endangered species impacts (USFWS 2022).

However, some participants cannot utilize these options:

“there are all these initiatives to help streamline this restoration work but the current laws are geared for straight restoration. And most projects nowadays, it’s […] multi benefits. You’re not just doing restoration for restoration.” -- R40

Some state-level streamlining does not apply to all projects, particularly large multi-benefit projects with non-restoration components. Increasingly, wetland restoration is completed alongside flood protection engineering such as new levees, or construction of transportation or recreation infrastructure (Camhi 2024; SLT n.d.). These may not fit habitat-only requirements of available streamlining. The conception of restoration projects by policymakers and regulators (as solely habitat projects) does not match that of project developers (as multi-sector, multi-benefit projects). As the nature of restoration shifts over time, policy must adjust. Regulators and policymakers should reevaluate current streamlining policies to identify if adjustments are needed, particularly an expansion of the types of projects or actions covered under streamlining policy language.

Participants also experienced conflicting permit requirements as a regulatory barrier. When multiple agencies with different missions grant approvals, accommodating all requirements within a single project design may not be feasible. Participants want more flexibility to manage conflicting regulatory requirements; as one said:

“it would be really helpful to give the agencies some more room to make some tradeoffs in time, some tradeoffs between species. Sometimes there are winners and losers. But when something is overall gonna have a really fantastic, overall positive effect” -- R11

Some efforts aim to increase coordination among regulatory agencies for restoration approvals. The Bay Restoration Regulatory Integration Team (BRRIT) convenes staff from six regulatory agencies to conduct a single, coordinated pre-application process for restoration projects in the Bay (BRRIT 2023). Further, individual projects have negotiated with regulators for in-lieu provision of certain requirements off-site when project designs or site conditions cannot accommodate all objectives. One participant described developing off-site recreation to avoid on-site safety concerns:

“bordered another […] property that is operated as a duck club. So the people eventually agreed that that would be too intrusive to have the public trail in an area where there was private management and hunting going on.”

Such efforts still involve lengthy, costly negotiations on a project-by-project basis. Desired wetland restoration outcomes in regional policy and regulation often conflict on-site, necessitating negotiated compromises between benefits in implementation (Gmoser-Daskalakis 2025). Shifting to a regional regulatory vision for wetland restoration outcomes at the landscape scale could enable more flexibility within individual projects without compromising overall social and environmental outcomes. Not every potential restoration outcome can be optimized, and some outcomes are reduced when adding other components (Tan et al. 2022). Rather than each project meeting multiple permit conditions, such as sensitive habitat and recreation, requirements could be evaluated and met across the full portfolio of projects in the region. One site may accommodate more public access and another more sensitive habitat. Rather than achieving this via multiple individual permits for each project, landscape-scale assessment of benefit provision across projects could achieve regional goals while reducing project-level negotiations.

Such a solution may generate conflicts. Regulators or stakeholders may be concerned about further streamlining or flexibility compromising goals of specific regulations, such as the Endangered Species Act. Further, each project site involves stakeholder goals from project partners, landowners, funders, and the public that may be difficult to reconcile or narrow down (Gmoser-Daskalakis 2025). But the idea of integrated permitting to create flexibility across multiple permits without compromising environmental outcomes has long existed in the literature (Rabe 1995). NbS literature also recommends regional planning with more focus on landscape conditions and interdependencies to address benefit synergies and trade-offs (Albert et al. 2019; van Rees et al. 2024). Creating a satisfactory regional regulatory vision for wetland outcomes requires extensive collaboration and alignment between regulatory actors, and scientists must understand how projects interact to produce landscape-level outcomes. Existing regional partnerships and scientific research institutes could expand their efforts to connect Bay and Delta practitioners, scientists, and regulators (SFEP, n.d.; SFEI, n.d.; DSC 2024). More collaboration must also address related regulatory barriers, particularly with municipal actors and utilities.

### Building Collaboration with Local Actors

Despite efforts to consolidate state or federal permits, projects still need easements from local governments, special districts, or even utilities and adjacent landowners, which increases costs and delays. Restoration in highly-developed estuaries like the Bay-Delta requires more local approvals, as available parcels are often located by or within multiple land uses and authorities, including utility infrastructure. Requirements for these approvals sometimes conflict with other regulatory requirements. Easements are context-specific to each project site and influenced by the goals of local actors. Participants report local-level approvals often require relationships built on a project-by-project basis, which can be costly or create delays:

“working on land with a ton of landowners, a ton of infrastructure and different easements that are needed […] We made great progress on [a prior] project […] We haven’t gotten there with [this] project with them [Utility], and that has been a challenge. […] It will happen eventually, but it might take a long time, and it’s already taken a long time.” -- R30

Further, participants find approvals often come from city attorneys or legal teams that lack understanding of restoration or do not regularly collaborate with project partners:

“You’re overlooking […] essentially veto power in the hands of some city or county administrator […] they can just grind everything to halt. […] And there’s almost no effort put towards greasing those wheels a little bit” -- R7

“Often in counties or municipalities, these decisions about permitting are really made largely through their counsel, their attorneys, not through people who do natural resource management, and in their planning departments. The planners are largely around the human, built, gray infrastructure […] habitat restoration is largely unknown.” -- R19

Broader, systematic efforts at collaboration, information sharing, and training with local governments, urban planners, utilities, and infrastructure operators could ensure these actors better understand restoration needs and prevent the need to restart negotiations with every project. Social-ecological systems literature recommends trusted organizations connect interdependent but otherwise disconnected actors to increase collaboration (Sayles and Baggio 2017; Vantaggiato et al. 2023; Wiegant et al. 2022; Tait and Brunson 2022; Rozance et al. 2020; Adams et al. 2021). Multiple participants recommended Resource Conservation Districts (non-regulatory special districts who work with landowners and local governments on environmental management) and Joint Ventures (voluntary regional partnerships for migratory bird protection) to reach local actors for education and relationship-building.

### Dedicated Project Stewardship Funding

Funding was the second most reported barrier subtype; participants said funding often comes with expiration dates, restrictions, or fluctuations subject to political will or state revenues. Participants specifically lacked funding for certain project phases. At present, growing state revenues, state bonds, and federal funding bills have increased public funds for wetland restoration (SFBRA 2025; NOAA 2025; Petek 2026). However, some funds, particularly state bonds, must be spent on ‘shovel-ready’ capital projects or construction phases, with less funding available for planning, design, pre-construction site management, and long-term, post-construction monitoring and maintenance. Participants struggled with obtaining sufficient funds for site stewardship and long-term maintenance from funders:

“it’s not easy, but it’s possible to find money to actually do the restoration. But it’s really hard to find the money to continue to do maintenance […] that’s not a priority out there.” -- R2

“It’s very difficult to get funding for any long term operations.[…] it’s still underfunded and just anything that needs to be operated like water control structures, or […] weeding […] patrolling a site that’s open to the public or not […] that has been very hard to come by.” -- R11

Literature already recommends expanding the timing of funding for estuary management (Sayles and Baggio 2017), blue-green infrastructure (Deely et al. 2020), and restoration (Sapkota et al. 2018). Private funders are suited to target the full project cycle, given legal limitations that prevent some public funds like bonds for non-capital investments (CA Govt Code § 16727 2025). Ecosystem restoration literature calls for more private funding, including in long-term monitoring (Lofqvist & Ghazoul 2019; Ermgassen & Lofqvist 2024; Biasin et al. 2024). Foundations and nonprofits may have more flexibility to alter their funding requirements and allocations. Several participants used private grants to fill project funding gaps for planning, design, site preparation, monitoring and maintenance. One private funder already recognizes their role as ‘gap filler’, specifically for pre-construction phases:

“I look at our role as not funding the actual restoration, say, or the construction, the earth moving, but enabling restoration. So that means funding early stage, getting these projects to the point where they can get permitted […] where they can put [in] an application for significant public funds.” -- R35

Adjusting funding guidelines or creating new funding sources to target site maintenance can address this barrier, but may require cognitive shifts in how funders think about project timescales and their role in restoration, expanding beyond construction and ‘ribbon cutting’. One participant found the motivation of funders overemphasized construction compared to maintenance:

“a lot of funders don’t want to fund anything but the construction cause that’s the dramatic piece, right? They want to fund something that you change and then walk away from, which can’t be the case.” -- R32

Similarly, NbS studies of green stormwater infrastructure and urban parks recognize insufficient funder and policymaker prioritization of long-term maintenance (Dempsey & Burton 2012; Mell 2021; Knapick et al. 2025; WWF 2025). Studies recommend private actor funding, including through Corporate Social Responsibility, for green infrastructure maintenance (Mell 2021) and coastal restoration (Sanchez-Arcilla et al. 2022). Wetland restoration also needs more awareness among funders around the value of long-term maintenance and adjusted incentive structures to motivate funding these activities.

### Study Limitations and Future Applications

As climate adaptation increasingly incorporates on-the-ground actions like wetland restoration, scholars must consider how current perspectives of climate adaptation must shift. This study demonstrates climate adaptation barrier frameworks apply to wetland restoration implementation, but require adjustment to reflect physical conditions and timing of project processes. Consideration of spatial and temporal scales can diagnose barriers and identify solutions to accelerate wetland restoration. Our adjusted framework may apply to other adaptation interventions such as NbS for flooding and extreme heat (e.g., green stormwater infrastructure, urban forestry). It works well for regions or systems with significant urban development and complex socio-political institutions, as it includes social-ecological conditions and interactions between spatial and temporal scales.

However, our study has limitations for broader application. Notably, reported barriers were not hard limits or obstacles to project completion. We have no counterfactuals of projects that failed or never began. Identifying this universe of ‘failed’ projects may prove difficult but could illuminate limits to implementation. Recall bias is an additional concern inherent in interviews about past projects. Future research applying this framework to other restoration projects or NbS can determine the applicability of our findings beyond our cases.

## Conclusion

Our study shows wetland restoration implementation involves a complex array of physical and governance factors from project developers, regulatory structures, and land parcels. Increasing restoration to meet targets requires diagnosing barriers in the implementation process; current literature either lacks a generalizable framework or does not adequately incorporate site-level considerations. We propose a barriers framework that better incorporates project-scale realities of wetland restoration.

Applying our framework to the California Bay-Delta reveals project partners experience barriers from the built and natural environment, permitting, and funding. Efforts to reduce regulatory barriers must facilitate collaboration between restoration practitioners, policymakers, local governments, and utilities to better accommodate site-specific conditions in regulation. There may be a role for regional partnerships and non-regulatory organizations to play by connecting actors operating at different scales. Funders, particularly private funders, can target the full project cycle, especially for long-term maintenance.

Future research could apply this framework elsewhere; similar barriers may signal our recommendations are relevant to other developed, coastal estuaries promoting wetland restoration for climate adaptation. This research is a first step to more explicit examination of the social-ecological process of wetland restoration. We demonstrate wetland restoration implementation faces unique challenges, particularly in translating regional policies to proximate project realities. Approaches rooted in social-ecological systems can navigate the dual social-ecological nature of wetland restoration to facilitate the expansion of this climate adaptation strategy in California and globally.

## Supplementary information

SupplementaryMaterials_Clean_EM_R2

## Data Availability

Anonymized data and code to reproduce the analysis presented in this paper are available at https://github.com/ucd-cepb/Bay-Delta-Wetlands-Barriers.

## Footnotes

1 Certain agencies require project entries in EcoAtlas as a condition of permit approval or funding. Several regional planning entities encourage and support other project developers to voluntarily enter their projects in EcoAtlas. EcoAtlas managers enter historic projects into the database. Conversations with regional experts and EcoAtlas managers confirmed data quality and coverage is sufficient for sampling purposes.

2 Each contact received 3 emails (1+ week apart) until labeled non-respondent; of 11 non-respondents, nine never replied, two refused due to schedule constraints, and six declared themselves insufficiently related to the cases (four referred us to other staff who responded).

3 Three interviews were conducted with two people each when two staff members from the same organization worked together and were interviewed together.

4 The author looked for keywords such as ‘barrier’, ‘challenge’, ‘frustration’, ‘issue’, ‘problem’, ‘struggle’ in relation to the restoration process.

5 For regional actors (i.e., funders, policymakers, program managers) this question was,“What are the major barriers you experienced completing or supporting wetland restoration in the region?”.

6 Regional participants like policymakers or funders not tied to a specific case study had ‘regional’ as the project and a location of Bay or Delta based on their wetland work’s geographic focus.

7 Both assistants coded a training set of 114 barriers from the first 7 interviews then discussed with the first author to reach full agreement. They then coded a test set of 69 barriers from 7 randomly selected interviews, resulting in agreement of 80% for barrier types, 75% for subtypes, 75% for spatial/jurisdictional barrier origins, and 70% for temporal barrier origins.

8 The project process refers to tasks after initial project siting/conception, including project design, regulatory and legal approvals, community engagement, funding management, and physical construction such as levee breaches and planting.
