# Supplementary material for: Diagnosing Barriers to California Bay-Delta Wetland Restoration Implementation: Extending the Adaptation Perspective
Source: Environ Manage. 2026 Sep 11;76(9):308. doi: 10.1007/s00267-026-02618-7 (PMC13569567; doi:10.1007/s00267-026-02618-7)
Supplement: Supplementary file 1 [file 267_2026_2618_MOESM1_ESM.docx]

Supplementary Materials

**Online Resource 1 Shortcomings in Existing Barriers Literature Relevant to Wetland Restoration (Wetland Restoration, Nature-Based Solutions, Climate Adaptation Studies)**

|  | Studies | Topic | Value for Application to Wetland Restoration | Limitations for Application to Wetland Restoration |
| --- | --- | --- | --- | --- |
| General Frameworks | Biesbroek et al. 2013 | Climate Adaptation | General framework, Classifies types of barriers, Based on synthesis of adaptation studies | Does not capture multiple temporal or spatial scales, physical/ecological site conditions, or project implementation staff constraints |
|  | Moser & Ekstrom 2010 | Climate Adaptation | General framework, Classifies the origins of barriers temporally and spatially | Does not capture physical and ecological site conditions |
|  | Deely et al. 2020 | Blue-Green Infrastructure | General framework, Considers barriers from project site conditions | Does not capture multiple temporal or spatial scales, for blue-green infrastructure rather than wetland restoration specifically |
| Case-Specific Studies | Raska et al. 2022; Sanchez-Arcilla et al. 2022; Stewart-Sinclair et al. 2020; Tuihedur Rahman et al. 2021 | Coastal/Marine NbS and/or Restoration | Identify or classify barriers to habitat restoration or management | Individual barrier classification systems are developed for each study (not generalizable) or for specific ecosystem types (coastal/marine, forest, riverine) or infrastructure (types of NbS like blue-green infrastructure) rather than a replicable framework for wetland restoration projects |
|  | Van Rees et al. 2024; Schultz et al. 2019 | Other Restoration (River, Forest) |  |  |
|  | Bataille et al. 2023; Bell-James 2022; Clare and Creed 2022; Sayles 2018; Tait and Brunson 2021 | Wetland Restoration or Management |  |  |

**Online Resource 2 Table of Case Study Projects**

|  | Location | Years | Habitat/ Size | Notable Features |
| --- | --- | --- | --- | --- |
| A | Delta | 2003- Present | 1,187 Acres | - Ongoing research partnerships  - 3 parcels in 2 phases  - Mitigation project on former farmland |
| B | Bay (Outer Coast) | 2000- 2008 | 550 Acres | - Nonprofit as lead partnered with government  - Voluntary project on former farmland  - Ongoing monitoring, public events  - Public outreach during construction |
| C | Bay | 1996-2014 | 700 Acres | - Facilitated beneficial reuse of sediment  - Repurposed military property  - Mix of seasonal and tidal wetland habitats |
| D | Bay | 2005-2011 | 4,500 Acres | - Restoration of a mix of habitat  - Repurposed industrial property  - Unanticipated levee breaches and engineering design challenges |
| E | Bay | 2016-  Present | 550 Acre Restoration Component | - Flood protection and SLR with habitat  - Ongoing community engagement by several community organizations |
| F | Bay | 2003- Present | 15,100 Acres | - Has Adaptive Management Plan  - Repurposed industrial property  - Restoration of a mix of habitat  - Ties in with larger shoreline flood protection project (with levee investments) |
| G | Bay | 2004- 2016 | 960 acres (2,327 total site) | - Site bought and restored by land trust before being transferred to government  - Voluntary project on former farmland  - Engineered post-construction design responses  - Transportation infrastructure altered designs |
| H | Delta | 1997- 2017 | 1950 Acres (307 + 600+ 300 + 740 + 3) | - Five separate projects on former farmland  - Carbon sequestration, subsidence reversal, habitat enhancement goals  - Ongoing research partnerships |
| J | Delta (Suisun Marsh) | 2011-2019 | 420 Acres | - Meets targets for Suisun Marsh Plan and Endangered Species Act compliance  - Former managed wetland for duck hunting converted to tidal wetland  - Challenges with Invasive Plants  - In-Lieu Public Access for Permits |

**Online Resource 3 Semi Structured Interview Script**

Part 1: Wetland Restoration Experience

1. What is your official job title? What tasks do you perform in your position in relation to wetland restoration? For example, planning, project management, engineering, advocacy/ outreach/ engagement, funding, or permitting.

2. How long have you worked in wetland restoration? How many wetland restoration projects have you worked on?

3. How has your organization been involved in wetland restoration in the [San Francisco Bay or Sacramento San Joaquin Delta]? For example, is your organization involved in any of the following: research, project design/management/construction/monitoring, advocacy/outreach/engagement, funding or permitting?

3A. How many years has your organization been involved in wetland restoration?

Part 2: Specific Wetland Restoration Project

I’d now like to discuss your organization’s experience with a specific wetland restoration project that you were involved in: the ____(Insert Case Study Project Here)________

[Regional: ‘wetland restoration you’re involved in’]

*Facts of the Project*

4. Could you tell me more about the history of ___(Insert Case Study Project Here)___? What led to the project being developed?

4A. What factors determined the specific location of this project?

[Question skipped for regional actors]

5. What were the main benefits you anticipated for the project? Which of them were a priority for your organization?

5A. Has your perception of the benefits of the project changed over time (the types or amount)?

[Regional: ‘for wetland restoration’]

6. Who do you understand receives the benefits of this restoration project? Or, who do you believe is the target population for this project’s benefits?

6A. Has your understanding of the project’s beneficiaries changed over the lifespan of the project? If so, how?

[Regional: ‘of wetland restoration’]

7. How do you think about this project in terms of climate adaptation and mitigation benefits? Has that changed over the lifespan of the project?

7A. Has there been discussion of carbon sequestration benefits from this project?

Do you consider carbon sequestration benefits in wetland restoration projects you work on?

[Regional: ‘wetland restoration’]

8. Did this project meet any goals or priorities of any local, regional, or state climate mitigation, climate adaptation, or development plans? If so, which?

8A. Did this[these] plan[s] support the planning and/or implementation of this project?

[Regional: ‘does wetland restoration’, ‘do these plans support planning or implementation of wetland restoration’]

Part 3: The Collaborative Process

Next, I would like to ask you about your experience with collaboration on this project [Regional: wetland restoration]. In particular, I’d like to discuss the organizations you partnered with on this project [Regional: most often for wetland restoration]. Partner refers to an organization with which you had regular collaboration for the implementation of the project such as through funding, regulatory requirements, design, construction, community engagement, monitoring etc. Partner organizations can include government agencies, nonprofits, community groups, private firms, foundations, etc.

*Project Partnerships*

9. What organizations did you partner with on this project? How and why did you select these partners to work with?

9A. What roles did your organization and each of your partners play on the project? For example, planning, project management, engineering, advocacy/ outreach/ engagement, funding, or permitting.

[Regional: ‘most frequently partner with for wetland restoration’]

10. Were community members or community-based organizations involved in the project? If so, how?

10A. How were community interests integrated into the planning or implementation of the project?

10B. What motivated that? Was there a funding or policy mandate for engagement or was it an organization decision?

[Regional: ‘in your work with wetland restoration’]

*Project Barriers*

11. What were the major barriers you experienced on the project?

[Regional: ‘completing or supporting wetland restoration in the region’]

12. How are you monitoring and evaluating the success of the project?

12A. How do you track implementation over time and different partners’ ongoing commitments to the project?

[Regional: ‘wetland restoration policy/goals’]

Part 4: Thank You!

Thank you for your time and involvement in this interview. Your answers have been invaluable to help me understand wetland restoration implementation in the region.

13. We’ve covered a lot of topics today, is there anything crucial we missed about the project?

[Regional: ‘wetland restoration in the region’]

14. Is there anyone else at your organization or another stakeholder organization you feel would be a good person to give information about____(Insert Case Study Project Here)______? [Regional: information about wetland restoration in the Bay or Delta]

**Online Resource 4 Test Statistics and Significance for Chi-Squared and Fischer’s Exact Tests for Differences in Reported Barrier Types and Origins by Location, Project, and Role Type**

Differences in Barrier Types by Project Location in Bay or Delta (See Online Resource 5A for Frequencies)

Barrier Types by Bay or Delta Location Chi-Squared Test Statistic 9.964, p-value=0.076 (Not Significant at p<0.05)

Differences in Barrier Origins by Project Location in Bay or Delta (See Online Resource 5B for Frequencies)

Spatial/Jurisdictional Barrier Origins by Bay or Delta Location Chi- Squared Test Statistic 0.878, p-value = 0.349 (Not Significant at p<0.05)

Temporal Barrier Origins by Bay or Delta Location Chi- Squared Test Statistic 4.957, p-value=0.084 (Not Significant at p<0.05)

Differences in Barrier Types by Project (See Online Resource 6 for Frequencies)

Barrier Types by Project Fisher’s Exact Test p-value=0.001 (Significant at p<0.05)

Test Statistics for Differences in Barrier Origins by Project (See Online Resource 7 for Frequencies)

Spatial/Jurisdictional Barrier Origins by Project Chi-Squared Test Statistic 44.160, p-value = 1.319x10^-6^ (Significant at p<0.05)

Temporal Barrier Origins by Project Fisher’s Exact Test p-value = 0.2155 (Not Significant at p<0.05)

Differences in Barrier Types by Staff Role Type (See Online Resource 8 for Frequencies)

Barrier Types by Project Chi-Squared Test Statistic 27.081, p-value=0.003 (Significant at p<0.05)

Test Statistics for Differences in Barrier Origins by Staff Role Type (See Online Resource 9 for Frequencies)

Differences in Spatial/Jurisdictional Barrier Origins by Staff Role Type Chi- Squared Test Statistic 6.999, p-value = 0.0302 (Significant at p<0.05)

Differences in Temporal Barrier Origins by Staff Role Type Chi- Squared Test Statistic 4.8122, p-value=0.3071 (Not Significant at p<0.05)

**Online Resource 5 Barrier Categories and Origins by Bay or Delta Location**

**5A. Reported Barrier Types by Bay or Delta Location**

|  | Bay (n=468) | Delta (n=361) |
| --- | --- | --- |
| Cognitive | 39 (8.3%) | 39 (10.8%) |
| Informational | 49 (10.5%) | 55 (15.2%) |
| Institutional | 92 (19.7%) | 71 (19.7%) |
| Physical/Environmental | 121 (25.9%) | 98 (27.1%) |
| Resources | 84 (17.9%) | 52 (14.4%) |
| Social | 83 (17.7%) | 46 (12.7%) |

**5B. Reported Barrier Origins by Bay or Delta Location**

|  | Bay (n=468) | Delta (n=361) |
| --- | --- | --- |
| Spatial/Jurisdictional | Proximate- 255 (54.5%)  Remote- 213 (45.5%) | Proximate- 184 (51.0%)  Remote- 177 (49.0%) |
| Temporal | Legacy- 72 (15.4%)  Contemporary- 341 (72.9%)  Future- 55 (11.8%) | Legacy- 61 (16.9%)  Contemporary- 240 (66.5%)  Future- 60 (16.6%) |

**Online Resource 6 Reported Barrier Types by Project**

|  | A | B | C | D | E | F | G | H | J | Region |
| --- | --- | --- | --- | --- | --- | --- | --- | --- | --- | --- |
| Cognitive | 6 | 8 | 9 | 2 | 0 | 9 | 2 | 14 | 4 | 24 |
| Informational | 17 | 11 | 11 | 10 | 0 | 5 | 6 | 8 | 11 | 25 |
| Institutional | 17 | 6 | 25 | 9 | 5 | 23 | 10 | 10 | 10 | 48 |
| Physical/  Ecological | 25 | 14 | 37 | 21 | 0 | 21 | 17 | 33 | 8 | 43 |
| Resources | 19 | 11 | 8 | 6 | 5 | 15 | 13 | 10 | 6 | 43 |
| Social | 15 | 22 | 11 | 6 | 9 | 16 | 13 | 9 | 7 | 21 |

**Online Resource 7 Reported Barrier Origins by Project**

|  | Spatial/Jurisdictional | Temporal |
| --- | --- | --- |
| A  (N=99) | Proximate-57 (57.6%)  Remote-42 (42.4%) | Legacy-13 (13.1%)  Contemporary-71 (71.7%)  Future-15 (15.2%) |
| B  (N=72) | Proximate-56 (77.8%)  Remote-16 (22.2%) | Legacy-6 (8.3%)  Contemporary-59 (81.9%)  Future-7 (9.7%) |
| C  (N=101) | Proximate- 58 (57.4%)  Remote- 43 (42.6%) | Legacy- 22 (21.8%)  Contemporary- 68 (67.3%)  Future- 11 (10.9%) |
| D  (N=54) | Proximate- 35 (64.8%)  Remote- 19 (35.2%) | Legacy- 6 (11.1%)  Contemporary- 38 (70.4%)  Future- 10 (18.5%) |
| E  (N=19) | Proximate-7 (36.8%)  Remote-12 (63.2%) | Legacy- 1 (5.3%)  Contemporary- 14 (73.7%)  Future- 4 (21.1%) |
| F  (N=89) | Proximate-38 (42.7%)  Remote-51 (57.3%) | Legacy-13 (14.6%)  Contemporary-69 (77.5%)  Future-7 (7.9%) |
| G  (N=61) | Proximate-39 (63.9%)  Remote-22 (36.1%) | Legacy-11 (18.0%)  Contemporary-43 (70.5%)  Future-7 (11.5%) |
| H  (N=84) | Proximate-45 (53.6%)  Remote-39 (46.4%) | Legacy-11 (13.1%)  Contemporary-59 (70.2%)  Future-14 (16.7%) |
| J  (N=46) | Proximate-19 (41.3%)  Remote-27 (58.7%) | Legacy-7 (15.2%)  Contemporary-31 (67.4%)  Future-8 (17.4%) |
| Regional  (N=204) | Proximate-85 (41.7%)  Remote-119 (58.3%) | Legacy-43 (21.1%)  Contemporary-129 (63.2%)  Future-32 (15.7%) |

**Online Resource 8 Reported Barrier Types by Participant Role**

| Role | Barriers (Total, Percent of Barriers Reported by Participant Type) |
| --- | --- |
| Project/Program Manager (426 Barriers, 25 Participants) | Cognitive (40, 9.39%)  Informational (57, 13.38%)  Institutional (95, 22.3%)  Physical/Eco (107, 25.12%)  Resources (57, 13.38%)  Social (70, 16.43%) |
| Technical Staff (303 Barriers, 24 Participants)    [Biologist, Construction Manager, Engineer, Environmental Scientist, Ecologist, Hydrologist, Operations Manager] | Cognitive (27, 8.91%)  Informational (38, 12.54%)  Institutional (50, 16.50%)  Physical/Eco (95, 31.35%)  Resources (48, 15.84%)  Social (45, 14.85%) |
| Policy Actor (100 Barriers, 10 Participants)    [Community Engagement, Executive Director, Funder, Policy Coordinator, Politician] | Cognitive (11, 11%)  Informational (9, 9%)  Institutional (18, 18%)  Physical/Eco (17, 17%)  Resources (31, 31%)  Social (14, 14%) |

**Online Resource 9 Reported Barrier Origins by Participant Role**

| Role | Spatial/Jurisdictional | Temporal |
| --- | --- | --- |
| Project/Program Manager (426 Barriers, 25 Interviewees) | Proximate (228, 53.5%)  Remote (198, 46.5%) | Legacy (73, 17.13%)  Contemporary (300, 70.42%)  Future (53, 12.44%) |
| Technical Staff (303 Barriers, 24 Interviewees)    [Biologist, Construction Manager, Engineer, Environmental Scientist, Ecologist, Hydrologist, Operations Manager] | Proximate (170, 56.10%)  Remote (133, 43.89%) | Legacy (40, 13.20%)  Contemporary (214, 70.63%)  Future (49, 16.17%) |
| Policy Actor (100 Barriers, 10 interviewees)    [Community Engagement, Executive Director, Funder, Policy Coordinator, Politician] | Proximate (41, 41%)  Remote (59, 59%) | Legacy (20, 20%)  Contemporary (67, 67%)  Future (13, 13%) |
